# A Network Pharmacology Study on the Cervix Prescription for Treatment of Cervical Cancer

**DOI:** 10.1155/2022/8945591

**Published:** 2022-10-12

**Authors:** Yingping Zhu, Liangping Wang, Leilai Xu, Pian Ying

**Affiliations:** ^1^Department of Obstetrics and Gynecology, The First Affiliated Hospital of Zhejiang Chinese Medical University, Hangzhou, 310006, China; ^2^Affiliated People's Hospital of Hangzhou Medical College, Zhejiang Provincial People's Hospital, China

## Abstract

**Purpose:**

Based on the method of network pharmacology to explore the mechanism of the cervical prescription (CP) in the treatment of cervical cancer (CC).

**Methods:**

We obtained the active ingredients and potential targets in the CP from the literature and the systematic pharmacological analysis platform of traditional Chinese medicine (BATMAN-TCM); the database was used to search for targets related to cervical cancer and to map CP and targets; the core targets were screened, and the protein-protein interaction network (PPI) was constructed using the TCM compound-target network and STRING database. Gene ontology (GO) and Kyoto Gene and Genome Encyclopedia (KEGG) pathway enrichment analysis of overlapping targets were performed using DAVID 6.8 online tool.

**Results:**

The CP contains 2 active ingredients, corresponding to 301 nonreactive targets; 10 GO biological process related items and 73 signal pathways were obtained. Cell experiments confirmed that the medicated serum of CP could effectively inhibit the proliferation and invasion ability of Hela cells.

**Conclusion:**

This study provides valuable information for TCM researchers and clinicians to better understand the main therapeutic targets and therapeutic roles of herbal decoctions in clinical settings. The results of our study preliminarily clarified that the cervical prescription has an inhibitory effect on cervical cancer cells.

## 1. Introduction

Cervical cancer is the second most common malignancy that affects women, ranking after breast cancer [1]. There are about 75,000 new cases and 33,000 deaths in China each year, accounting for about 1/10 of the world's total cases [2]. Modern studies have confirmed that human papillomavirus (HPV) infection is closely related to the occurrence, development, treatment, and prevention of cervical cancer. At present, western medicine is mainly used for regular monitoring of HPV-susceptible and infected individuals to prevent and reduce the occurrence of cervical cancer. The treatment strategies of cervical cancer mainly includes surgery, radiotherapy, and chemotherapy. However, there is still a lack of treatment strategies for HPV infection in the cervix and vagina, and so effective treatment approaches should be determined [3]. By adjusting the immune function of the body, Chinese medicine has shown several irreplaceable advantages in improving the clinical symptoms of cancer patients as well as the quality of life of patients [4]. With the advancements in medical technology, the research on traditional Chinese medicine (TCM) in the field of antivirals is increasing. Recently, TCM has expanded its thinking on preventing and treating cervical cancer as well as cervical HPV infection.

TCM has the advantages of individualized treatment and multiple effective ingredients and multiple targets, with good developmental prospects. However, Chinese medicine therapy is like a black box, in which the specific mechanism and target of the drug's action remain unclear, hindering the improvement of its clinical efficacy.

Network pharmacology is suitable for characterizing multicomponent, multitarget, and multipathways of Chinese medicine. To further clarify the effectiveness of Chinese medicine compatibility in many aspects [5], this article adopted the method of network pharmacology and started with the idea of “molecular-target-pathway.” A network model with regard to components, targets, protein interactions, biological functions, and signaling pathways of “Cervical Recipe” was drawn, the potential relationship in the network system was analyzed, and the pharmacology methodology method was further clarified. This study provides valuable information for TCM researchers and clinicians to better understand the main therapeutic targets and therapeutic roles of herbal decoctions in clinical settings.

## 2. Materials and Method

### 2.1. Medicinal Herbs

Alum, catechu, and borneol are three traditional Chinese medicines. Cervical prescription (CP) mucilage: 3 g of cervical prescription, 0.5 g of astragalus gum, 3 mL of glycerin, and distilled water to 10 mL. The low dose is 5% (0.04 *μ*g/10 g), the middle dose is 10% (0.2 *μ*g/10 g), and the high dose is 15% (1 *μ*g/10 g). After the preparation of the cervical prescription, HPLC was used for quality control to ensure the identity of the formula.

### 2.2. Animals

Specific pathogen-free (SPF) healthy male Sprague Dawley (SD) rats, weighing 220 ± 30 g, were provided by Zhejiang University of Traditional Chinese Medicine (Production license: SYXK (Zhe)2021-0012) and were raised adaptively for 1 week, at a humidity of 50% and a temperature of 20–25°C with ad libitum access to food and water. All animal experiments were approved by the Animal Experiment Ethics Committee of Hangzhou HIBIO Technology/Co., Ltd. (license number: HB2005007).

### 2.3. Material and Reagents

Cervical cancer cell Hela: Cell Resource Center, Institute of Basic Medicine, Chinese Academy of Medical Sciences; DMEM medium: Gibco company, product number: C11995500BT; fetal bovine serum FBS, product number: 04-001-1ACS (BI company); penicillin sodium: product number: 69-57-8 (Dalian Meilun Biology); streptomycin sulfate: product no.: 3810-74-0 (Dalian Meilun Biology); other reagents are of domestic analytical grade.

### 2.4. Preparation of Medicated Serum for Cervical Prescription

Forty 8-week-old healthy male C57BL/6 J mice (about 25 g ± 3 g) were randomly divided into cervical prescription group and saline control group. The daily intragastric administration dose of mice was calculated according to the ratio of the clinical human daily dose to the human-mouse body surface area ratio. The administration was continued for one week. The eyeball was removed 2 hours after the last administration, and the blood was collected. After standing at room temperature for 2 hours, centrifuge at 1500 rpm for 25 min, suck the upper serum, filter and sterilize it, and then store it at -30°C for later use.

### 2.5. Analysis of Effective Ingredients Contained in Cervical Prescription

CP contains 30 g of alum, catechin, and 1 g borneol. The three medicines are mashed and mixed with sesame oil for use. The BATMAN-TCM database [6] was used to search these three Chinese medicines, found the compounds contained in them as effective ingredients, and deduplicated the effective ingredients in these Chinese medicines.

### 2.6. The Identification of Targets Related to Cervical Cancer

First, we used “cervical cancer” as the keyword in 5 databases, including in the Kyoto Encyclopedia of Genes and Genome (KEGG) database [7], PHARMGKB database [8], the Comparative Toxicogenomics Database (CTD) [9], GeneCards database [10], and DisGeNET database [11]. In “cervical cancer (CC),” the drug target molecules of cervical cancer can be obtained, we identified the intersection between disease targets and the predicted targets of the composition of CP, and the network was visualized using Cytoscape [12] (version 3.7.1) software.

### 2.7. Protein Interaction (PPI) Network Construction

To identify the roles of target proteins at the system level, genes related to the targets of CP in the treatment of cervical cancer were uploaded to the STRING database (https://www.string-db.org/) [13] to identify protein interactions. After obtaining the PPI network, the PPI network is visualized and analyzed using Cytoscape software. For larger reciprocal networks, the connectivity (degree) and betweenness of nodes were analyzed based on network statistics analysis using the cytohubba plug-in [14], and nodes with connectivity and betweenness greater than the average were screened as key targets for compounding.

### 2.8. GO Enrichment Analysis

This analysis used the Gene Ontology database [15] and KEGG pathway database [7] to perform functional enrichment analysis on the target gene list. A statistical algorithm (Fisher's exact test) was used to find out as to which GO TERM/pathway has the greatest correlation between a set of genes and the analysis results. Each item in the analysis results has a statistical value of *P* value to indicate its significance. The smaller the *P* value is, the higher the degree of enrichment of gene list in this GO TERM/pathway, and more likely the gene list affects the life activities of cells through this GO TERM/pathway, which deserves further study.

### 2.9. KEGG Pathway Analysis

To investigate the roles of the active ingredient targets of TCM in specific signal pathways, we used the DAVID V6.8 database to conduct pathway enrichment analysis using potential targets of CP for the treatment of cervical cancer; the pathways identified were considered to be drug-related pathways.

### 2.10. Survival Analysis

Select the target gene list, combine the clinical data and gene expression data in TCGA-CESC, and analyze the genes whose expression and prognosis are significantly correlated in this gene set. Using the median expression of the gene as the grouping boundary, the samples were divided into two groups with high expression and low expression, and the COX method was used to analyze the survival difference (overall survival difference and disease-free survival difference) between the two groups of samples, and make a survival curve. At the same time, the correlation between gene expression and other clinical parameters (such as case stage) in this list was also analyzed, and a forest plot was made.

### 2.11. Construction of a Composition-Target-Pathway Network

Cytoscape version 3.5.1 software was used to construct an in vivo composition-target-pathway network for CP. Cytoscape is an open-source biological information analysis software package; the core output of this software is a network showing edges between each node, thus representing interactions between the biological molecules involved.

### 2.12. Selection of Target Genes and the Verification of Pathways

Next, we obtained the intersection of the targets and pathways predicted by network pharmacology and transcriptomics sequencing; the results of this analysis were then used for verification experiments. The results of network pharmacology analysis are provided later in this article; the transcriptomic results were published in our previous article [16]. In total, there were several pathways in the intersection. The glucose metabolism and lncRNA ATB were chosen for verification due to their close association with cervical cancer.

### 2.13. Animal Model

HeLa cells were prepared into cell suspension, 5 × 10^6^ cells per mouse, 200 *μ*L per mouse was inoculated on the outside of the armpit of mice, and 24 mice were inoculated in total. The mean tumor volume reached approximately 100 mm^3^. According to tumor volume, they were randomly divided into 4 groups: control group, lentiviral lncRNA ATB shRNA group, lncRNA ATB overexpression vector group, and drug intervention group, with 6 animals in each group. Tumor volumes were weighed and measured 3 times a week. On the 14th day, after weighing the body weight and measuring the tumor volume, the tumor was dissected and photographed and weighed, and the tumor inhibition percentage (IR) was calculated for statistical analysis.

### 2.14. Immunohistochemistry

Tissues were harvested, fixed, and embedded in paraffin. The paraffin sections were then washed with xylene and dehydrated in a series of alcohol concentrations. Sections were then treated with citric acid antigen retrieval buffer (pH 6.0). After antigen retrieval, the sections incubated in 3% hydrogen peroxide (37°C, 25 min); 3% BSA was then dropped on to the sections to block nonspecific antibody binding. PBS and HRP were dropped successively on to the sections and then washed three times in PBS (pH 7.4) for 5 min per wash. Finally, the sections were stained in hematoxylin, dehydrated, and mounted for analysis.

### 2.15. RT-qPCR

Trizol reagent was used to extract total RNA from sample in accordance with the manufacturer's protocol. Then, 2 *μ*g of total RNA was used as a template for reverse transcription to synthesize cDNA. Quantitative fluorescence PCR (Thermo, American) was then used to determine the relative expression of LncRNA ATB, TGF*β*1, PPP6C, and miR-126.

### 2.16. Transwell Assay

Place the Matrigel in a 4°C refrigerator overnight to dissolve, dilute the Matrigel with serum-free cell culture medium to 300 *μ*L/mL at 4°C, take 100 *μ*L and spread it evenly on the top surface of the PET film of the cell culture tank, gently put the chamber into the well of a 24-well plate, place it at 37°C for about 3 hours, and take it out on a clean bench to dry overnight. The four kinds of stable bead cells were digested with 0.25% trypsin and centrifuged, resuspended in serum-free medium, counted, and the invasion was grouped at 2 × 10 4/well/density and plated in 24-well plates. Add 500-600 *μ*L of medium containing 10% serum to the chamber, and place it in a 37°C incubator overnight. After overnight incubation, washed 3 times with 1 × PBS, fixed with 4% paraformaldehyde at room temperature for 15 min, washed 3 times with 1 × PBS, wiped the upper chamber cells with cotton swabs, added crystal violet for 15 min, and washed with 1 × PBS 3 times, air-dried at room temperature, and photographed under a microscope.

### 2.17. Statistical Analysis

The data results were imported into SPSS 21.0 software, and the data were expressed as *x* ± *s*. One-way analysis of variance was used for processing. *P* < 0.05 indicated that the difference was statistically significant.

## 3. Results

### 3.1. Cervix Prescription Active Ingredients and Their Targets

Cervical prescription contains 3 TCMs, 2 were obtained from the BATMAN-TCM database, in which alum is not recorded in the BATMAN-TCM database, and sesame oil as a preparation is not recorded in the BATMAN-TCM database.

Catechu contains 17 active ingredients, and 8 active ingredients had structural information. After screening by target interaction ≥20 points, there are 4 active ingredients in this, and these have a total of 36 nonrepetitive interaction targets.

Borneol contains 32 active ingredients, and 17 active ingredients had structural information. After screening for target interaction ≥20 points, there are 15 active ingredients in borneol, and these have a total of 272 nonrepetitive interaction targets. Combining the active ingredients of all TCMs in cervical prescription (with ≥20 points of active ingredients that interact as targets) and interactive targets, 17 kinds of nonrepetitive active ingredients and 301 nonrepetitive interactive targets were obtained. For specific statistics, see Table 1.

**Figure 1:**
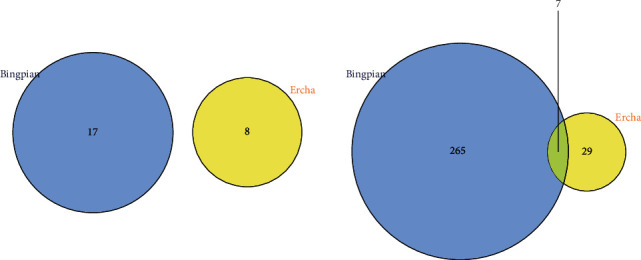
Interaction target intersection and union Venn diagram.

Cervical prescription contains 19 nonrepetitive active ingredients that have ≥20 points of mutual targets, and there are a total of 301 nonrepetitive drug targets. The intersection of all active ingredients in cervical prescription and their mutual targets was as follows (Figure 1).

All the active ingredients and target interaction genes were combined after screening for TCMs, and deduplication was performed to obtain nonrepetitive effective ingredients and nonrepetitive effective targets. The corresponding relationship between TCM and effective ingredients and the corresponding relationship between effective ingredients and target were then observed.

### 3.2. Analysis of Cervical Cancer Related Targets

In the KEGG and PHARMGKB databases, there are 7 target molecules for cervical cancer. In the CTD database, there are 1,658 target molecules obtained for cervical cancer, and there are 1211 target molecules obtained for cervical cancer from the GeneCards database. In the DisGeNE database, there are 964 target molecules obtained for cervical cancer.

**Figure 2:**
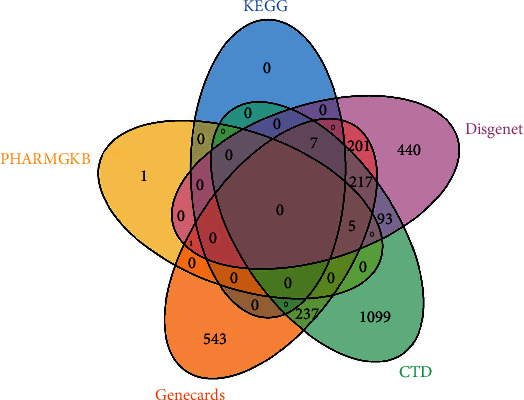
Five potential cervical cancer targets in databases.

The cervical cancer target molecules from the above 5 databases were combined to obtain 2,844 nonrepetitive cervical cancer target molecules. The intersection set of cervical cancer targets from the 5 databases was as follows (Figure 2).

### 3.3. Traditional Chinese Medicine-Ingredients-Target Analysis

**Figure 3:**
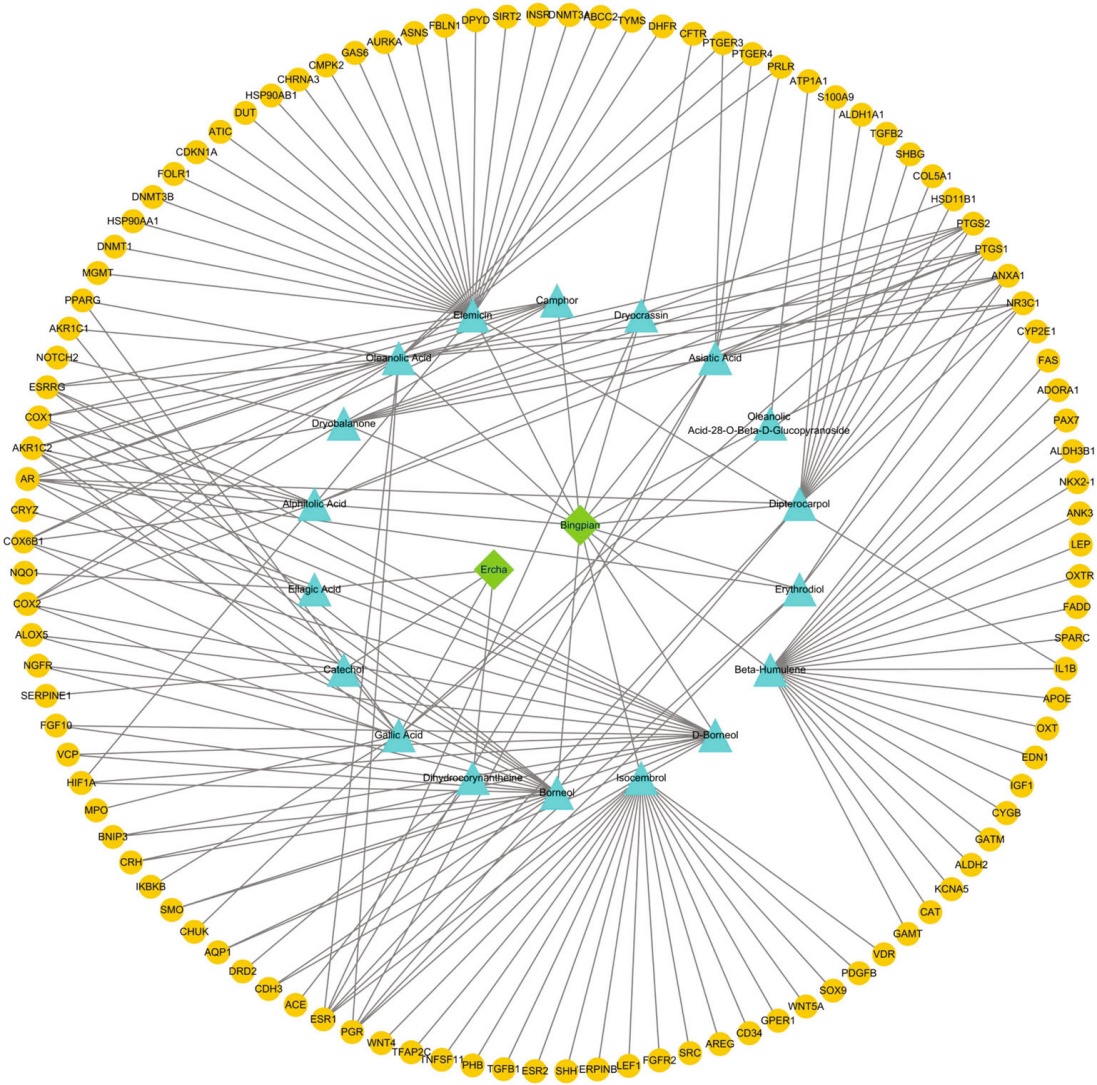
Traditional Chinese medicine compound-ingredients-target network.

The Chinese medicine-active ingredients and active ingredients-targets were combined to form a “Chinese medicine compound-components-targets” network and combined with the cervical cancer target molecules in 3.2 for further screening, and cytoscape was used to visualize the following (Figure 3).

### 3.4. PPI Network Analysis

**Figure 4:**
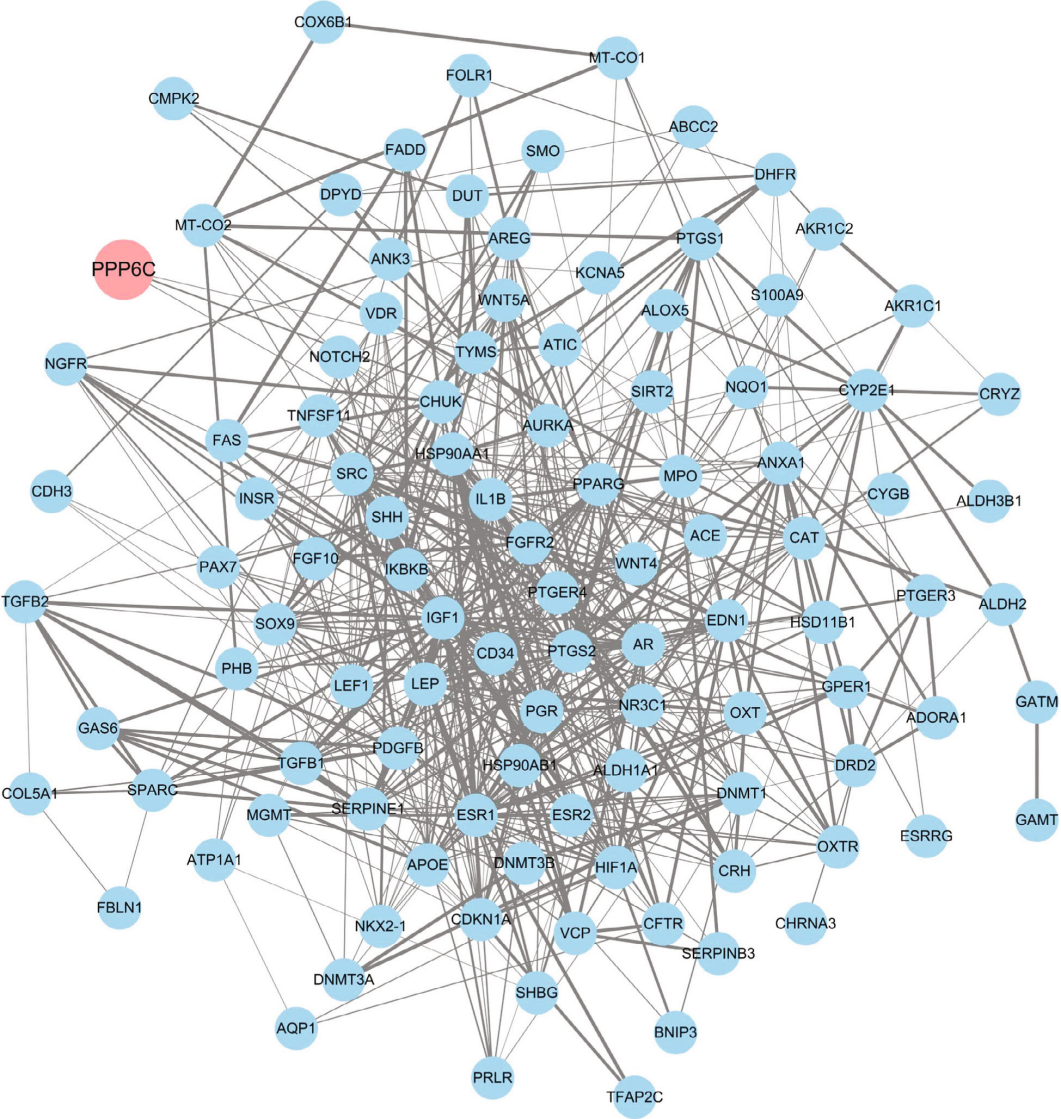
PPI interaction network of cervical cancer target protein.

For the target genes in the traditional Chinese medicine compound-component-target network, the STRING database was used to analyze the PPI interactions to obtain the interaction pair. The Cytoscape software was used to visualize it to obtain the following PPI network that has 106 nodes/proteins and 646 edges/interactions (Figure 4).

**Figure 5:**
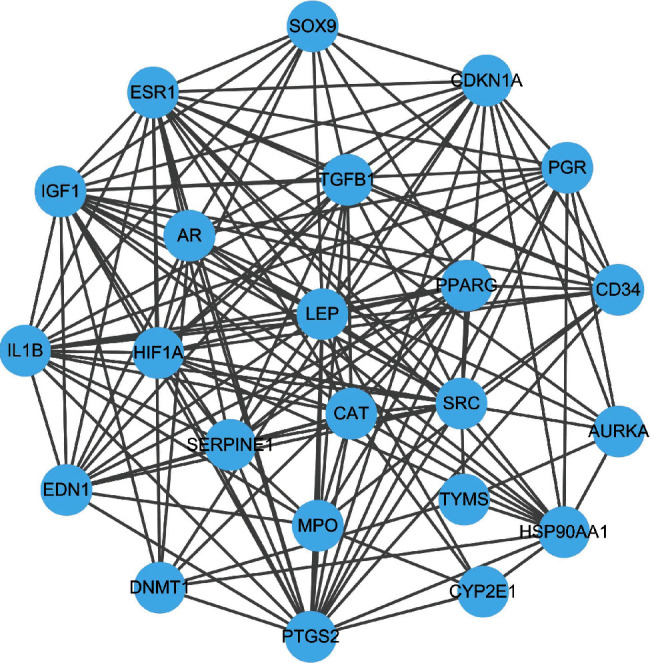
PPI interaction network of key target proteins of the compound.

The nodes whose connectivity and mediation degree are greater than the average value in the abovementioned PPI interaction network are screened as key targets of cervical prescription, and 23 key targets were obtained. The 23 targets constitute the following PPI interaction network, which has a total of 23 nodes/proteins and 147 edges/interactions (Figure 5).

### 3.5. Functional Enrichment Analysis

**Figure 6:**
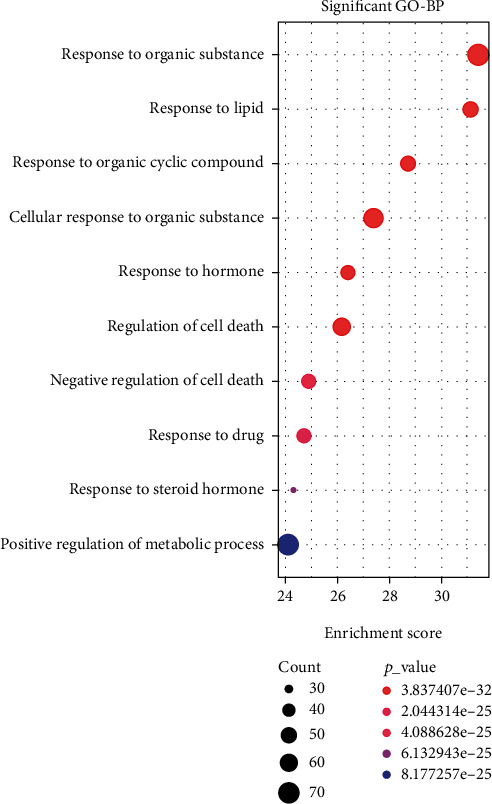
GO enrichment analysis of genes in the network of “Chinese medicine compound-components-targets”: bubble chart.

**Figure 7:**
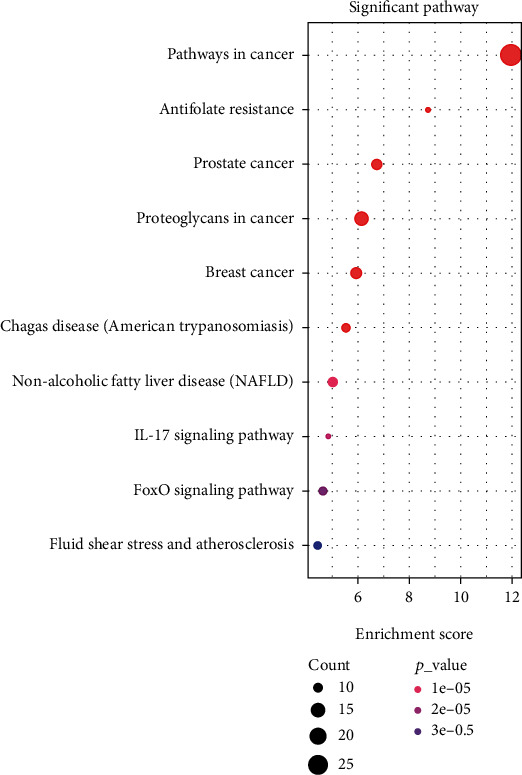
KEGG enrichment analysis of genes in the network of “Chinese medicine compound-components-targets”: bubble chart.

The functional enrichment analysis on genes (plus PPP6C) was performed in the “Chinese medicine compound-component-target” network to analyze their enrichment in GO ontology and KEGG and obtain the following enrichment results (Figures 6 and 7).

### 3.6. Survival Analysis

**Figure 8:**
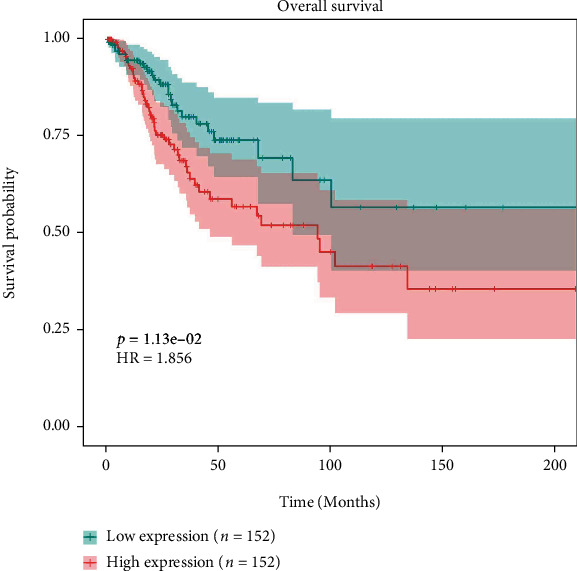
Difference analysis of overall survival rate of SERPINE1. It is worth mentioning that the disease-free survival period of the high and low expression groups of PPP6C gene can basically be regarded as a significant difference.

**Figure 9:**
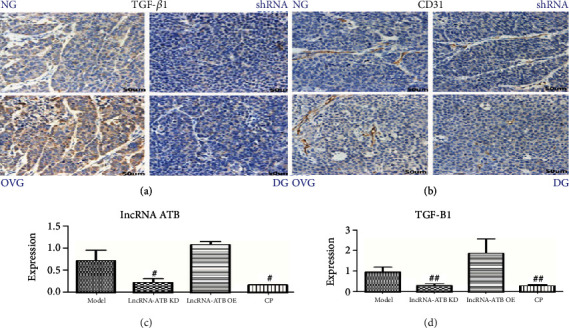
Immunohistochemical results: observed under the microscope, the positive was brown or yellowish brown. lncRNA-ATB promotes the growth of cervical cancer in vivo and the inhibitory effect of CP on lncRNA-ATB (c), TGF-*β*1 (a, d), and CD31 (b).

Survival analysis of 23 key target genes was performed using the clinical data and mRNA expression data obtained from the TCGA-CESC project, respectively. The results showed that the expression of 0/4 genes has significantly affected the disease-free survival rate/overall survival rate. An example of gene survival difference analysis was as follows (Figures 8 and 9).

### 3.7. Effects on HeLa Cell Growth Inhibition

Different concentrations of medicated serum of cervical prescription were used on Hela cells for 24 hours. The results showed that with the increase of drug concentration, the inhibition rate of Hela cells increased significantly (*P* < 0.05, *P* < 0.01), indicating that the medicated serum of Jinggong recipe It has obvious inhibitory effect on Hela cell growth, and the higher the concentration, the more obvious the effect of inhibiting cell growth (Table 2).

### 3.8. Inhibition of HeLa Cell Invasion

**Figure 10:**
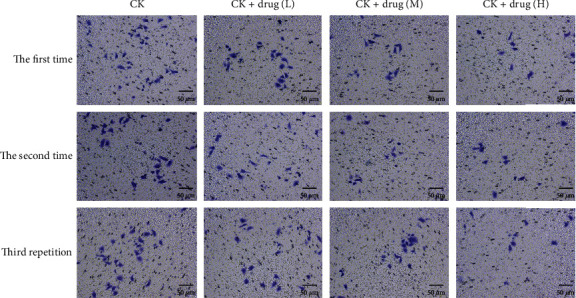
Cell statistics.

After 24 hours of different concentrations of the medicine-containing serum of the cervical recipe acted on HeLa cells, compared with the blank group, the cell invasion ability of the model group was significantly reduced (*P* < 0.01), indicating that cervical recipe has a significant inhibitory effect on the invasion ability of HeLa cells. And the higher the concentration, the more obvious the effect of inhibiting cell invasion (Figure 10).

### 3.9. In Vivo Tumor Growth Inhibition Experiment of Cervical Recipe

**Figure 11:**
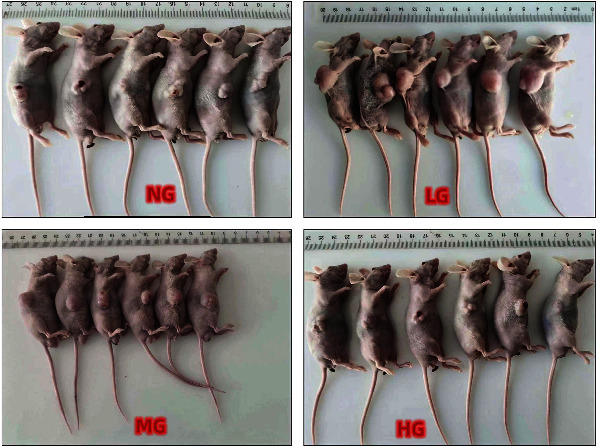
Inhibitory effect of cervical recipe on tumor-bearing mice.

24 male BALB/c nude mice, 8 w at the time of purchase, SPF grade, were acclimated to the animal room for one week before the test. The HeLa cells were prepared into a cell suspension, and 200 *μ*L per mouse was inoculated into the outer side of the axilla of mice according to 5 × 10^6^ cells per mouse, and the average tumor volume reached about 100 mm^3^. According to tumor volume, they were randomly divided into 4 groups: model control group (NG), low-dose traditional Chinese medicine group (LG), medium-dose traditional Chinese medicine group (MG), and high-dose traditional Chinese medicine group (HG), with 6 mice in each group. The model control group was given normal saline by gavage, the traditional Chinese medicine group was given 3 g of CP, and distilled water was added to make up to 10 mL. Dilute the cervical prescription liquid to 3 g/100 ml. Gavage: the low dose is 5% (0.04 *μ*g/10 g), the middle dose is 10% (0.2 *μ*g/10 g), and the high dose is 15% (1 *μ*g/10 g). After the preparation of the CP, HPLC was used for quality control to ensure the identity of the formula (Figure 11).

### 3.10. Statistical Analysis of the Antitumor Effect of Cervical Recipe In Vivo

**Figure 12:**
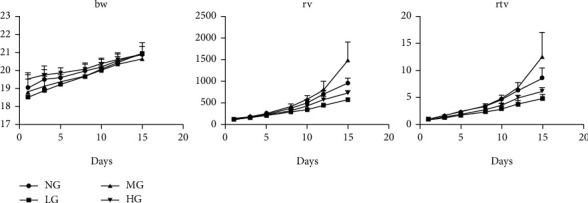
The reducing effect of cervical recipe on tumor weight and volume.

Tumor volumes were weighed and measured 3 times a week. After weighing the body weight and measuring the tumor volume on the 15th day, the tumor was dissected and photographed and weighed, and the tumor inhibition percentage (IR) was calculated (Figure 12).

## 4. Discussion

There is no record on cervical cancer and HPV infection in TCM, but the category of “carrying disease” and “sickness” can be attributed according to its clinical manifestations such as large amount and abnormal color and taste. “Carry down disease” involves a summary of abnormal diseases caused by vaginitis, cervicitis, pelvic inflammatory disease, and genital tumors in western medicine. TCM believes that cancer is an empirical evidence, and it is caused by damp heat and blood stasis toxins assault on the internal content, but cancer also includes internal deficiency. The pathogenesis of cervical cancer involves “lack of righteous qi, the damp heat and evil toxins congregating in the Zimen.” The most common type of cervical cancer syndrome differentiation involves damp toxins. The disease is located in any region of the cervix and the second vein. The treatment requires “clearing heat, detoxification, and attacking.” The most method used involves “dispersion of poison and strengthening the body.” The founder of cervical prescription, the Chief Physician Qiu Xiaomei, is in the first batch of national-level veteran traditional Chinese medicine practitioners and is the recipient of State Council allowances. He has been engaged in clinical teaching, treatment, and scientific research of Chinese medicine for more than 50 years.

According to many years of clinical experience, Doctor Qiu believed that the main cause of the disease involves dampness. Although dampness is a pathogenic condition, it causes invasion and transformation of dampness, but it lies in the imbalance of the liver, spleen, and kidney functions in the human body. All these pathologies and exogenous damp toxins might infiltrate and carry into the veins along the meridians. In the occurrence of cervical cancer, inflammation and damage of the cervical epithelium are the early stages of HPV infection and precancerous lesions.

The displacement of cervical squamous-column junction changes with the menstrual cycle and hormone levels, and the erosion-like changes in the transformation zone are almost considered inevitable. Therefore, how to ensure the integrity of the cervical epithelium is an important measure to prevent HPV infection and cervical cancer. For patients with cervicitis and HPV infection, and before they develop into cervical cancer, TCM with the philosophy of “preventing the disease before the disease should be applied to prevent the change of the existing disease.” Based on the syndrome differentiation and treatment, the combination of internal and external treatment can effectively control the condition. To improve the prognosis, the precancerous lesions of the cervix are mainly treated locally due to special location.

The external treatment of TCM not only involves surface absorption of effective ingredients of the medicine but they also can dredge the meridians and regulate the qi by stimulating the medicine to the specific part. This can be easily operated and is a relatively safe method for treating cervical cancer. No damage occurs to the gastrointestinal tract, and there is little impact on liver and kidney function. Catechu, borneol, alum, and other medicines when used in combination can promote local blood circulation and improve tissue metabolism in the diseased area, thereby weakening the local pathological reactions, eliminating discomfort, anti-inflammatory, analgesic, and reducing edema. In contrast, modern medicine uses laser, freezing, electric ironing, leap surgery, etc., which have certain advantages. After physical therapy and surgical treatment, vaginal discharge, watery discharge, irregular vaginal bleeding, and cervical scars might often occur postoperatively.

Cervical prescription is composed of alum, catechu, and borneol. The catechin is cool in nature, bitter in taste, and enters the heart and the lung meridians. According to the “Compendium of Materia Medica,” catechu is used for “coating golden sores, all kinds of sores, generating muscles and alleviating pain, stopping bleeding, and hygroscopic.” Modern pharmacological research showed that catechu contains catechin tannin, catechin, quercetin, and other ingredients. The main ingredient is the catechin tannin, which accounted for 20-50% of the catechu. It has inhibitory effects on Staphylococcus aureus, Candida albicans, Pseudomonas aeruginosa, and other bacteria and has certain analgesic and antitumor effects [17].

Borneol is slightly cold in nature, pungent in taste, and targets Guixin, spleen, and lung channels. “The Synopsis of the Golden Chamber” involves the use of borneol treatment for itching. The main ingredients of borneol include borneol and isoborneol. Tests have proved that borneol has obvious antibacterial effects on Staphylococcus aureus, Streptococcus, Escherichia coli, etc. [18].

Alum is sour, cold in nature, and used as astringent. It reaches to the lung, liver, spleen, and large intestine meridian. According to “The Classic”: “Alum is mainly for cold and heat to release diarrhea, white womb, erosive, eye pain, hard bones and teeth.” Alum mainly contains potassium sulphates. The tests showed that this product has an obvious inhibitory effects on a variety of Gram-positive bacteria. Alum liquid acts as a powerful astringent and causes damp-drying. When it meets the protein, it becomes a poorly soluble compound and then precipitates [19]. In this recipe, it mainly adheres to a moisture absorbent.

The key target network of cervical decoction was found in the treatment of cervical cancer, in which the TGF*β*1 signaling pathway, HIFa-1 signaling pathway, proteoglycan in cancer, and Th17 cell differentiation are considered as important gene targets, and these genes are all related to the regulation of immune system, which directly participates in tumor regulation pathway. GO analysis results showed that the biological processes involved in the enrichment of key gene targets include secretory granule lumen, cytoplasmic vesicle lumen, vesicle lumen, secretory granules, secretory vesicles, platelet alpha granule lumen, lysosomes, platelets alpha granule, cytoplasmic perinuclear cell, enzyme binding, sign receptor binding, heme binding, protein kinase binding, regulation of cell death, negative regulation of cell death, regulation of apoptosis process, regulation of programmed cell death, and procedural negative regulation of cell death.

It shows response to lipids, enzyme binding, organ-activated transcription factor activity, steroid hormone receptor activity, and nitric oxide synthase modulator activity. Enrichment analysis of KEGG pathway showed that the signaling pathways associated with cervical prescription in the treatment of cervical cancer include proteoglycan, HIF-1 signaling pathway, hsa01522pathview, hsa04114pathview, hsa04659pathview, hsa04914pathview, hsa04933pathview, hsa05200pathview, hsa05205pathview, and hsa05215 pathway. Studies have confirmed that hypoxia-inducible factor-1 (HIF-1), which is a highly specific nuclear transcription factor, plays an important role in the process of cell perception and adaptation to changes in internal environmental oxygen pressure [20]. Several studies have shown that HIF-1*α* has physiological effects such as promoting angiogenesis, regulating internal environmental pH, inducing autophagy and programmed cell death, and promoting self-renewability and differentiation of mesenchymal stem cells, and it is often found in a variety of primary. The expression level of HIF-1*α* in secondary malignant tumor tissues is shown to be abnormally increased [21]. The proangiogenesis function of HIF-1 is also involved in the remodeling of tumor microenvironment, indirectly promoting proliferation, metastasis of tumor cells and sensitivity to radiotherapy as well as chemotherapy [22].

In summary, this study found that cervical prescription mainly uses TGF*β*1, PPP6C, HIF1A, IL1B, and other gene targets through network pharmacology and regulates cancer pathways, FoxO signaling pathway, IL-17, and other signaling pathways to regulate immunity and improve cervical cell pathology in patients with cervical cancer, thereby exerting therapeutic effects of cervical cancer.

## 5. Conclusion

This study uses network pharmacology to explore the intervention of Qiu's Cervical Prescription on cervical cancer, which is reflected with the formation of cervical cancer, tumor cell metabolism, immune regulation, cell proliferation, apoptosis, and tumor resistance. During the early stage of cervical cancer, the formation of cervical cancer microenvironment can be adjusted to delay the carcinogenic process; and during the chemotherapy resistance period, the body hormones and tumor cell metabolism can be adjusted to increase tumor cell sensitivity and reverse the transformational therapy resistance. This also shows that the intervention of TCM for clearing heat and dampness and detoxification on cervical cancer is multitarget and multichannel and involves a systemic regulatory process. However, due to limitations in the conditions, this study also has many shortcomings, such as failed to integrate the concept of “junchen adjuvant” in TCM prescriptions, the impact of dose relationship in compound interventions in diseases, the lateness of data update, scientific research, the accuracy of network pharmacology, etc., leading to uncertainty of the prediction results of network pharmacology to a certain extent. So, the results should still be verified in a relevant basic research.

## Figures and Tables

**Table 1:** Compatibility of traditional Chinese medicine compounds-active ingredients-target statistics.

Chinese medicine	Number of active ingredients	Number of active ingredients (after screening)	Number of targets
Catechu	17/8	4	36
Borneol	32/17	15	272

**Table 2:** Cell number statistics table.

	NG	CP + LG	CK + MG	CK + HG
Repeat 1	24.60 ± 8.29	16.20 ± 4.09	13.40 ± 0.89	7.40 ± 1.14
Repeat 2	24.20 ± 7.26	18.40 ± 2.97	12.40 ± 1.82	8.20 ± 1.64
Repeat 3	24.40 ± 5.50	17.80 ± 3.56	10.40 ± 3.36	7.40 ± 1.82
Summary	24.40 ± 0.20	17.47 ± 1.14	12.07 ± 1.53	7.67 ± 0.46

## Data Availability

The data used to support the findings of this study are included within the article.
